# Outcomes of Image-Guided Moderately Hypofractionated Radiotherapy for Stage III Non-Small-Cell Lung Cancer

**DOI:** 10.1155/2021/2721261

**Published:** 2021-11-30

**Authors:** Yang Zhang, Zongjuan Li, Yixing Chen, Han Xiao, Yongkang Zhou, Shisuo Du, Zhaochong Zeng

**Affiliations:** Department of Radiation Oncology, Zhongshan Hospital, Fudan University, Shanghai, China

## Abstract

**Objective:**

To evaluate the efficacy and toxicity of hypofractionated radiotherapy (hypo-RT) for stage III non-small-cell lung cancer (NSCLC) in the Chinese population.

**Methods:**

Eighty-six stage III NSCLC patients who received hypo-RT (60 Gy/20 fractions, BED = 78.00 Gy: 73 patients; 62.5 Gy/25 fractions, BED = 78.13 Gy: 13 patients) were recruited. Fifty-seven patients who received conventional radiotherapy (60 Gy/30 fractions, BED = 72.00 Gy) during the same period were enrolled as the control group. All hypo-RT treatments were conducted using image-guided technology. The efficacy and toxicity of the treatment were compared between the two groups.

**Results:**

The median duration of follow-up was 23.0 months (range: 4.0–82.0 months). Univariate and multivariate analyses of all 143 stage III NSCLC patients revealed that hypo-RT was an independent factor for progression-free survival (PFS) and overall survival (OS). The median PFS and OS of hypo-RT were significantly higher than in the conventional RT group (PFS: 14.30, 11.00 months, *p*=0.035; OS: 43.30, 31.50 months, *p*=0.045). The incidence rates of symptomatic radiation pneumonitis and radiation esophagitis (≥grade 2) were 17.77% and 27.91%, respectively, in the hypo-RT group. Compared to the conventional radiation therapy group (22.81% and 19.30%, respectively), no significant differences were found between the two common side effects (*p*=0.662 and *p*=0.241, respectively).

**Conclusion:**

For Chinese stage III NSCLC patients, image-guided hypo-RT offers favorable prognosis, and the treatment toxicity was totally acceptable. This radiation modality deserves further prospective clinical trials.

## 1. Introduction

Radiation therapy (RT), with concurrent or sequential chemotherapy, remains the mainstream for locally advanced non-small-cell lung cancer (NSCLC). However, the outcomes under such a treatment strategy are not optimistic. By some estimates, local failure as the initial failure site occurs in approximately 35% to 40% of patients, indicating the need to intensify local-regional effects [1].

Improving the biologically effective dose (BED) received by patients is a potential treatment choice. In one study, a 1 Gy BED increase in the RT dose achieved an approximately 3% improvement in local control and a 4% improvement in survival [2]. However, the results of dose escalation from 60 to 74 Gy using conventional fractionation were detrimental [3]. A combination of several factors may contribute to this counterintuitive phenomenon, including accelerated tumor repopulation due to prolonged treatment time [4].

Moderately hypofractionated radiotherapy (hypo-RT) (2.3–5.5 Gy/fraction) is an alternative strategy to achieve higher BED without increasing cancer cell repopulation. Furthermore, hypo-RT shortens RT time, which could reduce the burden of medical institutions and increase the compliance of patients. In recent decades, technological advances, such as intensity-modulated RT (IMRT), helical tomotherapy, and image-guided RT (IGRT), have facilitated the use of this RT modality. Several clinical trials have revealed the preliminary results of the hypo-RT modality in NSCLC [5–11]. Although the prognostic outcome may be potentially promising, the exact role of moderately hypo-RT in NSCLC has not been validated through randomized phase III trials. In addition, only limited small-scaled moderately hypo-RT studies have been based on the Chinese population [12, 13].

The PACIFIC study has achieved tremendous accomplishments recently. With consolidation treatment using durvalumab after chemoradiotherapy in stage III NSCLC, up to 42.9% of patients achieved a 5-year survival [14]. However, the application rate of this novel treatment modality is currently not very high in China, mainly due to the elevated treatment costs. Thus, optimizing treatment modalities based on chemoradiotherapy still deserves attention. In addition, hypo-RT could effectively promote neoantigen generation, which could allow better cooperation with immunotherapy. Thus, exploring the application of hypo-RT in NSCLC has the potential to improve the therapeutic effects of the PACIFIC modality.

Therefore, in this study, we retrospectively enrolled a large cohort of stage III NSCLC who received moderately hypo-RT at our institute and compared the outcomes and toxicities of this RT modality with conventionally fractionated RT.

## 2. Patients and Methods

### 2.1. Patients

The Institutional Review Board of Zhongshan Hospital, Fudan University, approved this retrospective study. The enrollment criteria included the following: (1) pathological confirmation of NSCLC; (2) clinical stage III (American Joint Committee on Cancer Staging, 7^th^ version); (3) Eastern Cooperative Oncology Group (ECOG) performance status of 0-1; (4) no previous anticancer therapy; (5) received definitive moderately hypo-RT, with concurrent or sequential chemotherapy. In addition, patients who received conventional RT during the same period were also enrolled as the control group. The exclusion criteria were as follows: (1) concomitant diagnosis with other cancers or serious medical diseases with an adverse influence on prognosis; (2) lost to follow-up; (3) history of receiving any consolidation immunotherapy after chemoradiotherapy.

### 2.2. Treatment

Platinum-based doublet chemotherapy regimens were administered in NSCLC patients. For adenocarcinoma, pemetrexed combined with a platinum-based scheme was recommended, while gemcitabine or paclitaxel along with platinum was used in squamous cell lung cancer (gemcitabine was not administered concurrently with radiotherapy).

Radiation delivery was performed by IMRT or tomotherapy. The gross tumor volume (GTV) included all detectable carcinomas and lymph nodes with a short-axis diameter >1 cm visible on computed tomography (CT) imaging or positive features demonstrated by positron emission tomography-CT scans. The clinical target volume (CTV) of the primary tumor was determined using an isotropic 6–8 mm margin around the GTV. The CTV of the lymph nodes comprised the entire involved lymph node region detected by CT or positron emission tomography-CT. The planning target volume was delineated using a 1.0–1.5 cm margin around the CTV to compensate for breathing movement and setup errors. The objective of all RT plans was to deliver the prescribed dose to ≥95% of the planned target volume. The institutional dosimetry constraints for normal organs at risk (lungs, heart, esophagus, and spinal cord) were previously described in detail [15]. The following RT schemes were implemented: (1) 60 Gy/20 fractions (3.0 Gy/fraction, BED = 78 Gy, commonly used for peripheral tumors); (2) 62.5 Gy/25 fractions (2.5 Gy/fraction, BED = 78.13 Gy, commonly used for central tumors). In addition, patients who received 60 Gy/30 fractions (2.0 Gy/fraction, BED = 72 Gy) were also enrolled as the control RT regimen. Patients in the conventional and hypo-RT groups shared the same principles of treatment scheme, target volume delineation, and physical planning setup. Megavolt CT in tomotherapy was performed daily to ensure precise radiation delivery. Regarding IMRT, cone-beam CT was performed routinely for the first three days of RT and was then performed at least once a week.

### 2.3. Follow-Up

The patients were carefully monitored during the RT process and then followed up at regular intervals, approximately every 3 months for the first 3 years. Short treatment responses were evaluated according to the Response Evaluation Criteria in Solid Tumors (RECIST 1.1) [16]. The Common Terminology Criteria for Adverse Events version 4.0 were used to determine therapeutic toxicity. Progression-free survival (PFS) was defined as the time to local/systemic progression or the last follow-up. Overall survival (OS) was defined as the time of death from any cause or the last follow-up. PFS and OS were calculated from the start of treatment.

### 2.4. Statistics

Statistical analysis in this study was performed using R software version 4.0.5. Categorical variables in this study were shown as rates, and continuous variables were presented as mean ± standard deviation (normal distribution) or median + interquartile range (IQR) (abnormal distribution). The best cutoff values were calculated by receiver operating characteristic analysis. Response rates, radiation pneumonitis, and esophagitis were compared using the chi-square tests. Continuous variables were compared using independent Student's *t*-test (normal distribution) or nonparametric test (abnormal distribution). PFS and OS were estimated using the Kaplan–Meier method. Parameters significant in the univariate analysis were then included in the multivariate analysis using the Cox regression model. All statistical tests were two-sided, with significance defined as *p* < 0.05.

## 3. Results

A total of 143 stage III NSCLC patients were ultimately enrolled in this study. Among them, 86 received moderately hypo-RT (60 Gy/20 fractions: 73 patients; 62.5 Gy/25 fractions: 13 patients), and the other 57 participants were treated with conventional RT (60 Gy/30 fractions). The median follow-up period was 23.0 months (range: 4.0–82.0 months).

### 3.1. Survival Analysis for Stage III NSCLC Receiving Chemoradiotherapy

Univariate and multivariate analyses were performed to explore prognostic factors in all 143 stage III NSCLC patients. On univariate analysis, age (*p*=0.016), pathology (*p*=0.007), and RT modality (*p*=0.034) were identified as risk variables for PFS. After multivariate analysis, older age (*p*=0.020), nonadenocarcinoma pathology (*p*=0.019), and conventional RT (*p*=0.021) remained negative prognostic factors (Table 1). Regarding OS, tumor stage (*p*=0.041), pathology (*p*=0.010), and RT modality (*p*=0.040) were initially demonstrated to be related factors. After multivariate analysis, nonadenocarcinoma pathology (*p*=0.012) and conventional RT modality (*p*=0.046) remained adverse prognostic factors (Table 2). For the other variables, no impact on PFS and OS was found.

### 3.2. Comparison of the Conventional and Moderately Hypo-RT Modality

The baseline characteristics of the two treatment groups were compared. As shown in Table 3, no significant differences were identified in patient- and treatment-related clinical variables.

**Figure 1:**
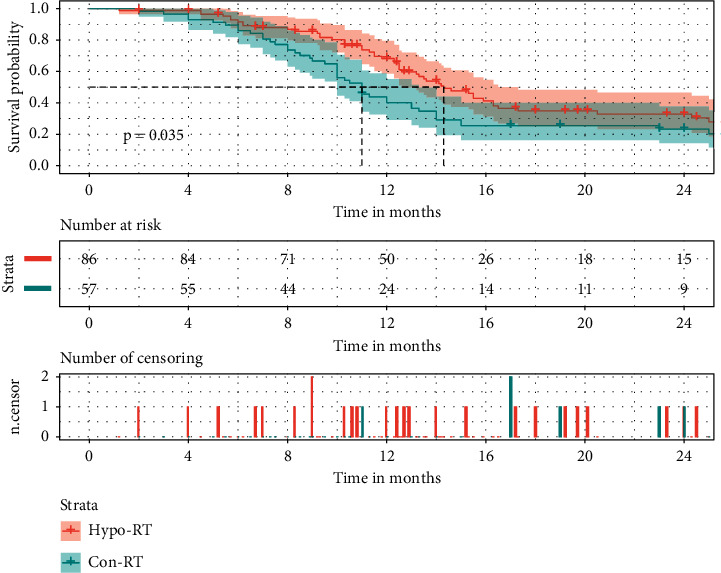
The effects of the RT modality on PFS in stage III NSCLC. PFS: progression-free survival; Hypo-RT: hypofractionated RT; Con-RT: conventional RT.

**Figure 2:**
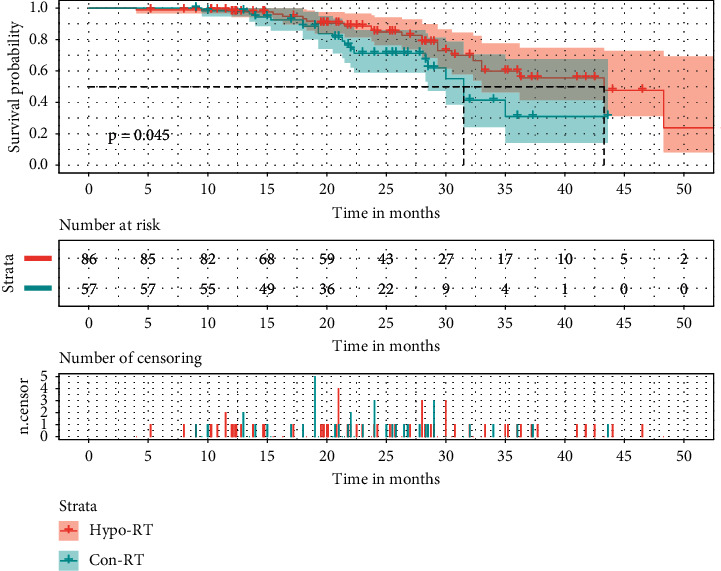
The effects of the RT modality on OS in stage III NSCLC. OS: overall survival; Hypo-RT: hypofractionated RT; Con-RT: conventional RT.

Further Kaplan–Meier analysis was conducted to compare the prognosis of the two different RT modalities. Compared to conventional RT, both median PFS and OS of the hypo-RT group were significantly higher (PFS: 14.30, 11.00 months, *p*=0.035, Figure 1; OS: 43.30, 31.50 months, *p*=0.045, Figure 2). In addition, no differences were found in the rates of response, symptomatic radiation pneumonitis, and radiation esophagitis between the above two groups (64.91% vs. 62.79%, *p*=0.796; 22.81% vs. 19.77%, *p*=0.662; 19.30% vs. 27.91%, *p*=0.241). In the conventional RT group, among the 42 progressive people, 59.5% were local-regional, 30.95% of patients had distant metastases only, and 9.5% had combined local-regional and distant recurrences. In the hypo-RT cohorts, 47.06% of failure patterns were local-regional, 43.14% of patients presented distant metastases as the initial progression site, and 9.80% had both local progression and distant recurrences.

### 3.3. Comparison of Concurrent and Sequential Chemoradiotherapy in the Moderately Hypo-RT Modality

**Figure 3:**
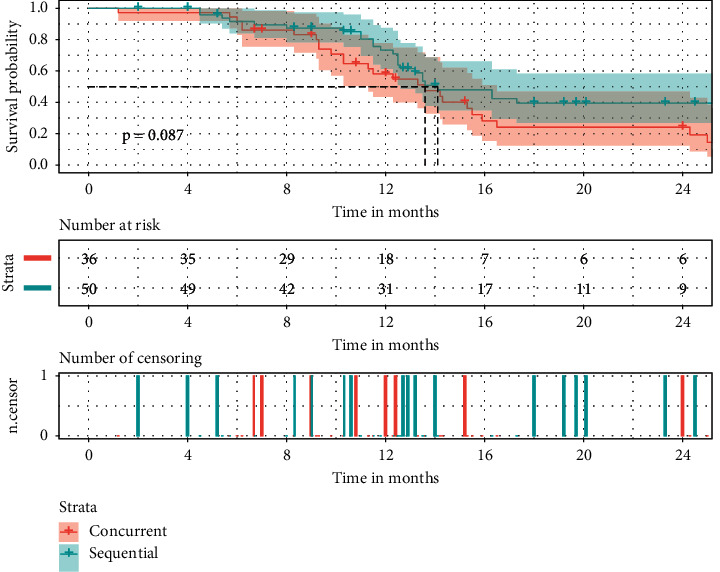
The effects of the chemotherapy type on PFS in hypo-RT-treated NSCLC. Hypo-RT: hypofractionated RT.

**Figure 4:**
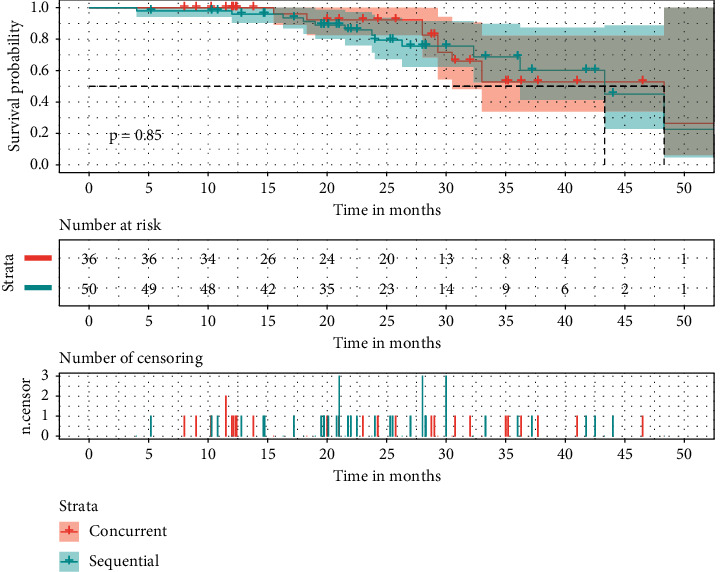
The effects of the chemotherapy type on OS in hypo-RT-treated NSCLC.

Concerns about the treatment toxicity partially impede the implementation of hypo-RT, especially under the context of concurrent chemotherapy. Therefore, we performed subgroup analysis according to the chemotherapy type in hypo-RT patients. After Kaplan–Meier analysis, no significant differences were seen in PFS (*p*=0.087, Figure 3) and OS (*p*=0.85, Figure 4). As for treatment response rate, symptomatic radiation pneumonitis, and radiation esophagitis also, no significant differences were shown (57.89% vs. 66.67%, *p*=0.403; 21.05% vs. 18.75%, *p*=0.790; 36.84% vs. 20.83%, *p*=0.100).

## 4. Discussion

NSCLC is a fast-growing tumor with a cell doubling time of only 2.5 to 3.3 days. RT duration longer than 6 weeks had an adverse effect on survival [17]. A dose-per-fraction escalation approach, namely, moderately hypo-RT (2.3–5.5 Gy/fraction), has been reported to achieve equivalent or even higher BED, without prolongation of RT time, thereby overcoming the effects of accelerated tumor proliferation. This is the major biological basis for the improved prognosis observed following hypo-RT. Furthermore, lymphocytes, acting as an important fighter of the immune system, have been demonstrated to be prognostic factors in NSCLC [18–21]. Our previous work revealed that hypo-RT was significantly associated with a decreased risk of severe lymphopenia; this may be another potential mechanism for the better prognosis achieved with hypo-RT [22].

The hypo-RT modality shows a trend of improved survival; however, there are concerns pertaining to RT toxicity [23]. Surely, in the context of three-dimensional conformal RT (3D-CRT), the probability of radiation pneumonia or esophagitis in NSCLC receiving hypo-RT was indeed higher [12, 24, 25]. Recently, along with the advent of IMRT and tomotherapy, delivering radiation more precisely to tumors while sparing normal organs has become possible [26–28]. In the present study, all patients received IMRT or tomotherapy; obviously, the toxicity was well tolerated. In addition to advanced RT plans, hypo-RT increases the accuracy requirements for the delivery of radiation [29]. IGRT is considered as a good strategy to address this issue and has been associated with a lower risk of radiation pneumonitis [30]. In our study, daily megavolt CT in tomotherapy was performed to ensure the precise delivery of radiation. Regarding IMRT, cone-beam CT was performed routinely for the first three days of RT and was then performed at least once a week. The above measures may explain the tolerable toxicity observed in this study.

With the success of the PACIFIC study, the landscape of stage III NSCLC is changing [31, 32]. Under such a treatment strategy, the rate of distant metastases was lower in the durvalumab group than in the placebo group [33, 34]. This underlines the importance of the local control. In our study, compared to conventional RT, the risk of local-regional failure in hypo-RT was relatively lower, indicating that hypo-RT may represent better cooperative partner with immune consolidation. In addition, under the PACIFIC treatment strategy, further reduction in treatment-induced toxicity, while maintaining optimal tumor control, has become a priority, thereby warranting more patients with access to consolidated immunotherapy. In this study, patients with sequential chemotherapy could achieve a similar prognosis compared to concurrent chemotherapy with the modality of hypo-RT. Similar with our results, Maguire et al. conducted a randomized phase II trial comparing sequential versus concurrent chemotherapy and radical hypo-RT in patients with inoperable stage III NSCLC and good performance status. They also revealed that prognosis of sequential chemoradiotherapy was not inferior to that of the concurrent modality [35]. Therefore, we propose that, in the context of “PACIFIC,” for moderately hypo-RT, sequential chemotherapy may be a better choice than concurrent chemotherapy; however, this needs to be confirmed by future clinical trials.

Iyengar et al. performed a randomized clinical trial which compared hypo-IGRT and conventional RT for patients with stage II/III NSCLC and poor performance status [36]. All participants in the hypo-RT group received daily IGRT, which may be the reason no differences in toxicity could be observed between the two treatment groups. As for prognosis, however, they revealed that the OS resulting from hypo-RT was not superior or even inferior to that of conventional RT. Firstly, as demonstrated in the paper, subsequent systemic therapy was an important factor affecting OS. However, only 26% of patients in the hypo-RT group received systemic therapy, and the rate increased to 37% in the conventional RT group. Secondly, the hypo-RT group had a larger proportion of patients older than 80 years.

Although our study has certain clinical significance, there are still several limitations to be considered. The principal shortcoming is the nature of the retrospective study design, which has led to some bias. Although our study had the largest sample size of the Chinese population to date, the homogeneity of the data collected had certain defects, including chemotherapy type and RT physical plan; however, this also gave us the opportunity to perform subgroup analysis and identify more significant associations. Furthermore, subsequent salvage treatments surely exert a significant influence on patient outcomes, especially in the era of targeted therapy and immunotherapy. Due to the nature of our retrospective observational study, subsequent treatment modalities were variable and could not be controlled. We were unable to conduct further prognostic analysis in this study. Thirdly, with the protection of IGRT, hypo-RT could be performed safely in some patients with central tumors. However, in cases where the tumors are very close to vital organs, such as the esophagus or heart, conventional radiotherapy may be a better option.

In conclusion, for Chinese stage III NSCLC patients, compared to conventional RT, image-guided hypo-RT with chemotherapy may lead to better prognosis and with well-tolerated treatment toxicity. Prospective multicenter randomized controlled studies should be conducted to further confirm our findings.

## Figures and Tables

**Table 1:** Univariate and multivariate analyses of PFS in the whole 143 stage III NSCLC patients.

Characteristics	Univariate analysis			Multivariate analysis		
	HR	95% CI	*p*	HR	95% CI	*p*
RT modality						
Hypo-RT	1			1		
Con-RT	1.55	1.03–2.32	0.034	1.62	1.07–2.43	0.021
Age						
<65	1			1		
≥65	1.69	1.1–2.58	0.016	1.66	1.08–2.56	0.020
Gender						
Male	1					
Female	0.68	0.33–1.4	0.291	NA		
Tumor stage						
IIIB	1					
IIIA	0.68	0.33–1.4	0.291	NA		
PS						
0						
1	1.17	0.78–1.76	0.436	NA		
Pathology						
Adenocarcinoma				1		
Nonadenocarcinoma	1.82	1.17–2.82	0.007	1.70	1.09–2.64	0.019
Chemotherapy type						
Concurrent						
Sequential	1.31	0.87–1.99	0.198	NA		

PFS: progression-free survival; NSCLC: non-small-cell lung cancer; RT: radiation therapy; Hypo-RT: hypofractionated RT; Con-RT: conventional RT; PS: performance status.

**Table 2:** Univariate and multivariate analyses of OS in the 143 stage III NSCLC patients.

Characteristics	Univariate analysis			Multivariate analysis		
	HR	95% CI	*p*	HR	95% CI	*p*
RT modality						
Hypo-RT	1			1		
Con-RT	1.89	1.15–3.57	0.040	1.87	1.20–3.56	0.046
Age						
<65	1					
≥65	1.49	0.76–2.94	0.249	NA		
Gender						
Male	1					
Female	0.51	0.12–2.11	0.351	NA		
Tumor stage						
IIIB	1			1		
IIIA	0.50	0.26–0.97	0.041	0.57	0.29–1.12	0.103
PS						
0	1					
1	0.86	0.46–1.58	0.618	NA		
Pathology						
Adenocarcinoma	1			1		
Nonadenocarcinoma	2.75	1.27–5.95	0.010	2.7	1.24–5.87	0.012
Chemotherapy type						
Concurrent	1					
Sequential	1.11	0.60–2.05	0.743	NA		

OS: overall survival; NSCLC: non-small-cell lung cancer; RT: radiation therapy; Hypo-RT: hypofractionated RT; Con-RT: conventional RT; PS: performance status.

**Table 3:** Patient characteristics in the conventional and hypo-RT treatment groups.

	Level	Con-RT, *n* (%)	Hypo-RT, *n* (%)	*p*
n		57 (39.86)	86 (60.14)	
Age	<65	27 (47.4)	34 (39.5)	0.450
	≥65	30 (52.6)	52 (60.5)	

Gender	Female	4 (7.0)	9 (10.5)	0.685
	Male	53 (93.0)	77 (89.5)	

Tumor stage	IIIA	37 (64.9)	47 (54.7)	0.295
	IIIB	20 (35.1)	39 (45.3)	

PS	0	29 (50.9)	50 (58.1)	0.494
	1	28 (49.1)	36 (41.9)	

Pathology	Adenocarcinoma	18 (31.6)	35 (40.7)	0.353
	Squamous cell carcinoma	39 (68.4)	51 (59.3)	

Chemotherapy modality	Concurrent	22 (38.6)	36 (41.9)	0.830
	Sequential	35 (61.4)	50 (58.1)	

PET-CT planning	Yes	24 (42.1)	39 (45.3)	0.702
	No	33 (57.9)	47 (54.7)	

PTV (cc), median [IQR]		121.5 [69.0, 190.5]	114.0 [67.5, 178.1]	0.759

Hypo-RT: hypofractionated RT; Con-RT: conventional RT; PS: performance status; PTV: planning target volume; IQR: interquartile range.

## Data Availability

The datasets used or analyzed during the current study are available from the corresponding author upon reasonable request.
